# Adsorption mechanism and valency of catechol-functionalized hyperbranched polyglycerols

**DOI:** 10.3762/bjoc.11.92

**Published:** 2015-05-18

**Authors:** Stefanie Krysiak, Qiang Wei, Klaus Rischka, Andreas Hartwig, Rainer Haag, Thorsten Hugel

**Affiliations:** 1 Physik Department and IMETUM, Technische Universität München, 85748 Garching, Germany; 2 Department of Chemistry and Biochemistry, Freie Universität Berlin, 14195 Berlin, Germany; 3 Fraunhofer Institute for Manufacturing Technology and Advanced Materials (FhG IFAM), 28359 Bremen, Germany; 4 Institute of Physical Chemistry, University of Freiburg, Albertstraße 23a, 79104 Freiburg, Germany

**Keywords:** adhesion, atomic force microscopy, catechol, hyperbranched polyglycerols, valency

## Abstract

Nature often serves as a model system for developing new adhesives. In aqueous environments, mussel-inspired adhesives are promising candidates. Understanding the mechanism of the extraordinarily strong adhesive bonds of the catechol group will likely aid in the development of adhesives. With this aim, we study the adhesion of catechol-based adhesives to metal oxides on the molecular level using atomic force microscopy (AFM). The comparison of single catechols (dopamine) with multiple catechols on hyperbranched polyglycerols (hPG) at various pH and dwell times allowed us to further increase our understanding. In particular, we were able to elucidate how to achieve strong bonds of different valency. It was concluded that hyperbranched polyglycerols with added catechol end groups are promising candidates for durable surface coatings.

## Introduction

While underwater glues are still a challenge for industrial adhesive development, mussels, barnacles and numerous other animals and plants have found a way for strong, long-term adhesion to wet surfaces [1]. Wet hydrophilic surfaces are difficult to be wetted by glues since the adhesive competes with the surface water layer [2]. Mussels can easily adhere to hydrophilic metal oxides (e.g. ship hulls) or mineral surfaces such as rocks, even against large tidal forces. Studying the mechanism of how mussels adhere gives us the opportunity to adapt these principles for the development of industrial coatings and biomedical adhesives.

Mussels adhere to surfaces via their byssus, a bundle of filaments with adhesive plaque on the end [3–4]. They are made of proteins and contain no living cells. To understand their adhesive properties the proteins in the byssus were studied extensively by numerous groups. The *Mytilus edulis* byssus contains about 25–30 different proteins; however, the part that adheres to external surfaces, the byssal plaque, contains only 7–8. Of these, 5 are unique to the plaque [5–6], namely the *Mytilus edulis* foot proteins (Mefp) 2, 3, 4, 5 and 6. Directly at the contact area, mainly Mefp 3, 5 and 6 are found. Mefp 3 and 5 are rich in 3,4-dihydroxyphenylalanine (DOPA, 15–30 mol %) [7–8]. DOPA is formed by posttranslational modification of tyrosine. Mefp 6 is rich in cystein (11 mol %) [6]. It has been found that the DOPA in Mefp 3 and 5 adheres to the surfaces, while the cystein-rich Mefp 6 controls the redox balance and can keep interfacial DOPA in a reduced state [5,9]. The byssal plaque also shows strong cohesion through crosslinks. The cysteins can crosslink with DOPA and the oxidized DOPA (semiquinones) can crosslink via radical addition. Furthermore, crosslinking by iron chelate complexes of DOPA improves cohesion [10].

The adhesion of a single DOPA to metal oxides was studied with AFM force spectroscopy [11–13] and rupture forces of up to 1000 pN were measured. This is on the same order of magnitude compared to forces of around 1400 pN that have been measured for the rupture of covalent bonds [14–16]. Besides the strength of the bond, the most interesting feature is that DOPA-based bonds were found to be reversible: once broken they can form again [11,17–18]. This flexibility and action seem to be a general principle for the formation of strong and durable interphases in natural as well as artificial systems [19].

Finally, not only is DOPA itself a key to understanding the adhesive properties of blue mussels, but also to understanding the primary structure of the respective protein or peptides containing the DOPA [20]. This primary structure should promote strong bond formation and self-healing. Here we use a hyperbranched polyglycerol as a hydrophilic core with numerous DOPA (catechol) groups attached. A similar system has already proven to be advantageous for an antifouling coating on titanium oxide surfaces [21–22]. An added benefit of this system is that the oxidation of catechol to quinones makes crosslinking possible and allows for good cohesion between the layers of this material. Here we investigated the molecular details, valency and dynamics on how molecules with multiple DOPA groups adhere to surfaces.

## Results and Discussion

The publication by Lee et al. [11] sparked considerable interest and since then several research groups have published results of single molecule atomic force measurements of DOPA or DOPA-containing molecules on metal oxide surfaces. The published work shows large variations between <100 pN up to almost 1000 pN [11–13,23]. The reasons for this large variation in the results are unclear, which underscores how little is known about the nature of the interaction between the catechol group of DOPA and metal oxide surfaces.

**Figure 1:**
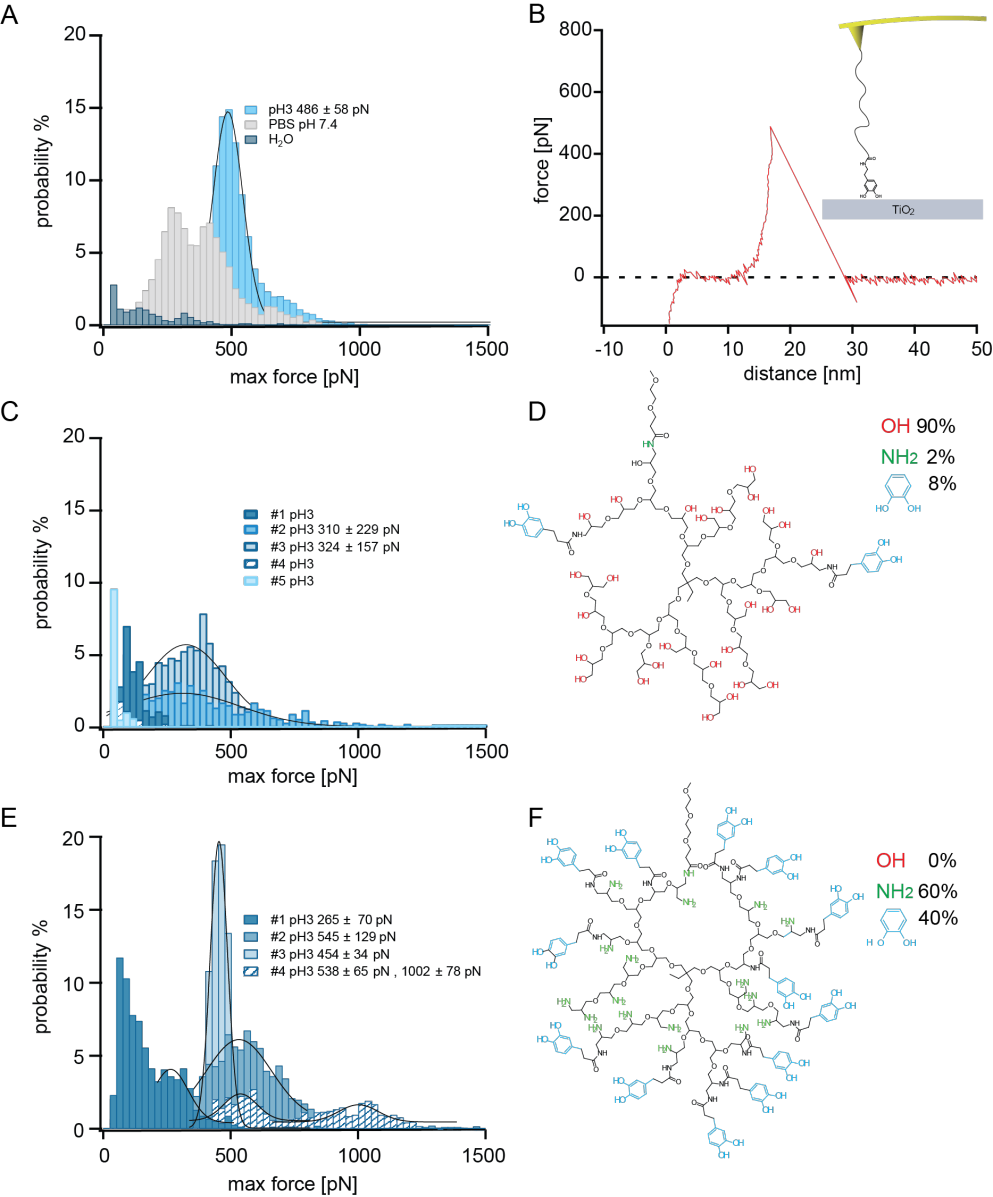
A) A maximum rupture force (max force) histogram for a dopamine-functionalized tip is given for the three measurement buffers: ultrapure water (dark blue), PBS (grey) and citric acid/phosphate buffer pH 3 (light blue). B) A typical retraction force–distance trace of the desorption of dopamine from TiO_2_. The inset shows a schematic of the dopamine desorption experiment. The dopamine is covalently coupled to the tip with a PEG linker using NHS ester chemistry and desorbed in buffer from TiO_2_. C) The max force histograms of 5 measurements of hPG with 8% catecholic end groups at pH 3 are depicted. D) The structure of the applied molecule. E) Max force histograms of 4 measurements at pH 3 of the hPG with 40% catechol end groups. F) The structure of the molecule utilized in (E), where possible peaks in the max force histograms were fitted and the peak value as well as the standard deviation are given in the insets.

To determine the force of a single catechol group on titanium dioxide, we performed AFM single molecule force spectroscopy measurements with tips functionalized with dopamine. Dopamine is derived from L-DOPA by removing the carboxyl group. This leaves an amine group that was used to covalently couple the probe molecule to the tip through a PEG linker using NHS-ester chemistry, as illustrated in the inset of Figure 1B. A sample force–distance trace showing the retraction of the tip from the TiO_2_ surface is shown.

The experiments were performed at room temperature with a constant pulling velocity of 1 µm/s and a surface dwell time of 1 s. Different buffers were used for the measurements. McIlvaines buffer solutions (a mix of 100 mM citric acid and 200 mM Na_2_HPO_4_) at pH 3 were used as well as phosphate-buffered saline (PBS) and ultrapure water. The maximum peak force as well as the detach force were extrapolated from the data.

Figure 1A shows the maximum detachment force (max force) of a dopamine-functionalized tip in ultrapure water, PBS and in citric acid/phosphate buffer at pH 3. The measurement in ultrapure water shows a very broad distribution of detachment forces with no distinct peak. There are small peaks at approximately 40, 140 and 320 pN as well as occasional high force events of up to 750 pN. The measurement of the same tip in PBS at pH 7.4 shows a clear bimodal force distribution with peaks at 290 pN and 410 pN and a shoulder at approximately 650 pN. For the same tip at pH 3, a high force peak at 500 pN with a shoulder at about 700 pN was measured.

Although the original measurement by Lee et al. [11] giving an average maximum detachment force of 805 pN was performed in water, our measurement in water showed no high force peak. Due to the lack of buffering capacity of ultrapure water, contamination could change the pH in unpredictable ways. This and the strong pH dependence of the high force interaction made it difficult to reproduce the measurement in ultrapure water. The measurement in PBS showed a bimodal distribution similar to the bimodal distribution measured in buffer of pH 8.3 in the publication by Lee et al. They attributed the high force peak (760 ± 90 pN) to unoxidized DOPA and the lower force peak (210 ± 70 pN) to oxidized DOPA–quinone. Similar to the measurement of Lee et al., the higher forces in our measurement occurred at the beginning of the measurement and the lower forces at the end. This is consistent with longer term oxidation as in the measurements by Wilke et al. [13]. An increasing pH shifted the equilibrium between DOPA and DOPA–quinone towards the oxidized quinone. While the lower force peak values were roughly comparable (290 pN vs 210 pN), our high force peak was considerably smaller than that of Lee et al. (410 pN vs 760 pN). The reasons for this could be differences in experimental parameters like the force loading rate or the surface dwell time or, even more likely, due to their valency, as discussed below. The measurements at low pH 3 showed a clear high force peak and a high probability of a desorption event. At this pH, one can be reasonably sure that the DOPA is not oxidized [24]. We attribute the 500 pN peak to the catechol/titanium dioxide interaction and the shoulder at 700 pN to the interaction of multiple catechols.

In a next step, the hyperbranched polyglycerols (hPGs) with different amounts of catechol end groups were desorbed from TiO_2_. The measurements were again performed with a pulling speed of 1 µm/s and a dwell time of 1 s in McIlvaines buffer at pH 3. The molecule with 8% catechol end groups is depicted in Figure 1D. Besides the 8% catechol end groups, most end groups (90%) are hydroxy groups. Of the five measurements depicted in Figure 1C, three showed mostly small maximum forces (below 200 pN) and occasional events at 200–300 pN. Two of the five measurements showed broad high force peaks (310 ± 230 pN, 320 ± 160 pN) containing events in the force range of the high force catechol–TiO_2_ interaction as well as events in the lower force range. The events in the lower force range could be due to the hydrogen bonds of the hydroxy end groups, which show forces below 200 pN (data not shown). One of the measurements showed occasional force events in the range of 700 pN to 1.2 nN, indicating that several catechols participated in the interaction. These could be either two catechol groups of one molecule or two molecules with catechol groups. The fact that only two of the five measurements showed high force peak interactions could be explained by geometrical constraints. The molecule was covalently attached to the tip by a PEG tether, which limits the ability of the molecule to rotate. With only 8% catechol, the possibility of interaction of the catechol groups with the surface depends on the position of the catechols relative to the tether. Figure 1D shows an example where it is unlikely for the catechol to interact with the surface. Since the position of the tether (coupled to an amino-functionalized site) and the catechols on the hPG is random, it will be possible to observe catechol–TiO_2_ interactions in some measurements and in others not.

The last molecule had a catecholic functionalization for 40% of its end groups and all other end groups were amino groups, as depicted in Figure 1F. The maximum force histograms are shown in Figure 1E. Three of the four measurements showed clear high force peaks at approximately 550, 450 and 540 pN. One showed a lower force peak at 270 pN that could indicate oxidation. Two measurements showed occasional events at even higher forces above 700 pN and another had a second high force peak at 1 nN. This is due to the interaction of two catechol groups, either multivalent by two catechols on one molecule or polyvalent by two molecules each with one catechol, as discussed below.

The measurement showed that adding more catechol end groups increases the likelihood of catechol–titanium dioxide interaction. Measurements of the molecule with 8% catechol showed high desorption forces for two of the five measurements, while for the molecule with 40% catechol, three of the four measurements showed high forces. Multiple catechol–titanium dioxide interactions were occasionally observed in one of the five measurements with 8% catechol and in two of four measurements with 40% catechol. Additionally, one of the four measurements of 40% catechol showed a clear second high force peak with forces corresponding to roughly twice the catechol–titanium dioxide desorption force. An increased adhesion caused by the additional amines would not lead to the observed narrow high force peak, but rather to a broad force peak with a tail towards lower forces.

**Figure 2:**
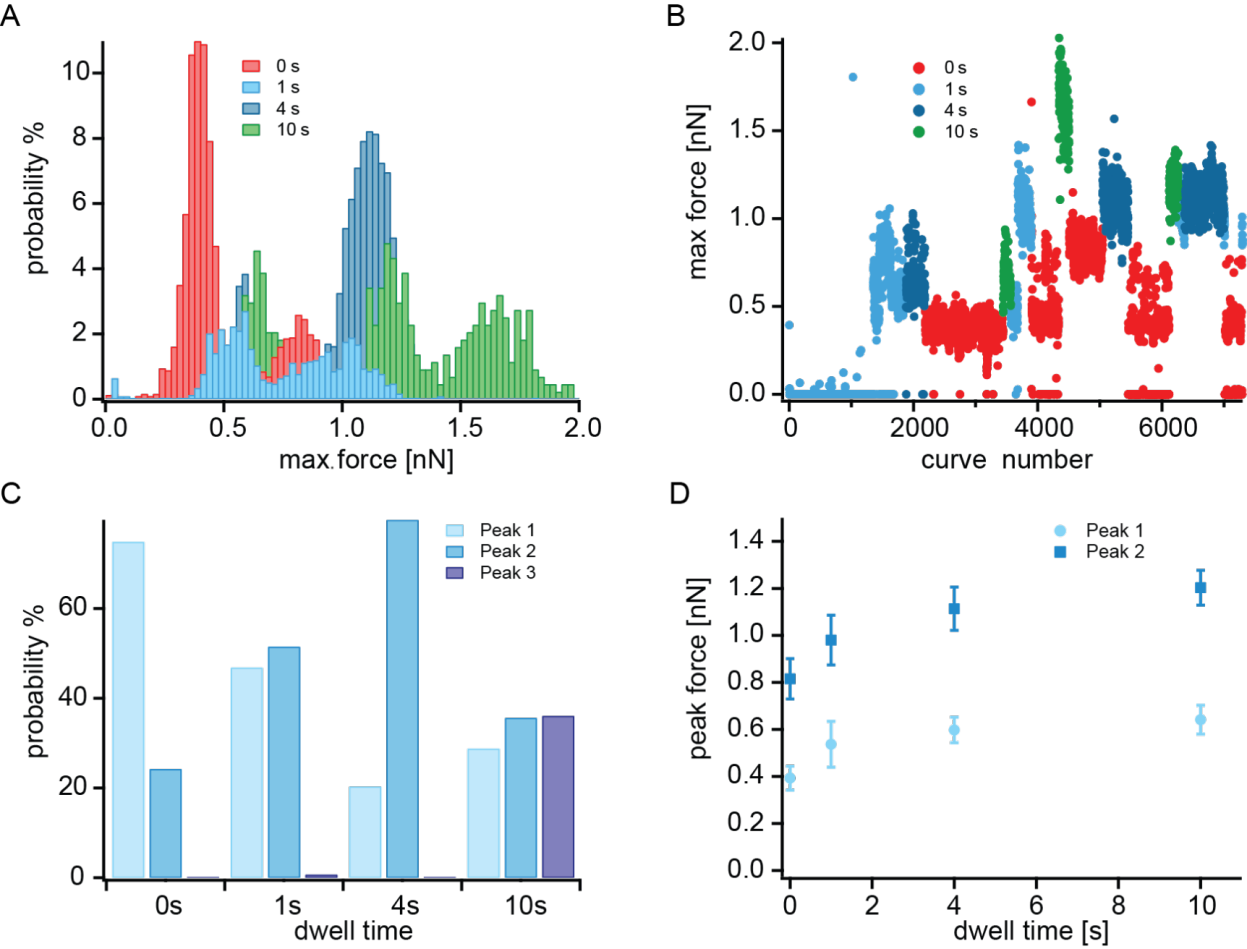
A) Maximum force histograms for the different dwell times indicated in the inset normalized to the number of measured force curves. B) Maximum force vs curve number is plotted to exclude bias due to the order of the measurement. C) Fraction of the number of force peaks belonging to poly- and/or multivalent interactions for the different surface contact times (see main text for details). D) Peak values of the maximum force histograms for the first and second peak are plotted against the dwell time. The standard deviation of the Gaussian fit is given as the error.

The measurements discussed thus far have been performed with a surface dwell time of 1 s. In the following the effect of the surface contact time on the probability and force of desorption is tested for hPG functionalized with 40% catechol. When considering surface contact time, not only must the dwell time at the trigger force value be considered, but also the time that is needed to reach the trigger force. Dwell times of 0, 1, 4 and 10 s were measured. In addition, at 0 s to the normal trigger force, a smaller trigger force was used as well. For the small trigger force, the tip needed 30 ms from the first surface contact to reach the trigger force and retract again until contact with the surface was lost. For the larger trigger force, this value was 160 ms. Thus the total surface contact time was 30 ms, 160 ms, 1.16 s, 4.16 s and 10.16 s. Each dwell time measurement was repeated in a different order to ensure that no time effect would obscure the result. Figure 2 summarizes the results of the measurement. In Figure 2A, the maximum force histograms associated with the different dwell times are plotted in different colors. The number of events is normalized to the number of measured force curves. The 0 s (red), 1 s (light blue), and 4 s (dark blue) dwell time measurements showed two clear peaks, each corresponding to interactions of one and two catechol groups with the titanium dioxide surface. The longest 10 s dwell time measurement had even three clear peaks. Two interesting conclusions can be drawn from the data. Multiple catechol events become more likely with increased dwell time, and interestingly, the single catechol interaction force increased with increasing surface contact time. Figure 2B shows the maximum force of each curve plotted against the curve number, where the data points are again color coded. This figure illustrates that the force does not change over time. The probability of observing an event was low for the first 1500 force curves (measured with a 1 s dwell time) and then very high until the end of the measurement. This might be due to conformational changes or the interaction of a different catechol unit. At the beginning of the measurement, the lowest average probability was 42% for the 1 s dwell measurement, because of the low overall probability of events. However, the 61% probability with the smaller trigger force and 30 ms contact time was not caused by the effect of time. This is a markedly lower probability compared to the 99.6% for the higher trigger force and 160 ms contact time. For the 4 s and 10 s dwell times, 99.8% and 100% of the curves showed events.

At the lower surface contact time, the first peak indicating a single catechol interaction is more prominent than the second peak. This behavior changes with increasing dwell time, as illustrated in Figure 2C. The probability of a single, double or triple interaction in relation to the total number of events is plotted for the different surface contact times. For 0 s dwell time, a single interaction was more probable. At 1 s dwell time, single and double events had a similar probability, and at 4 s the double catechol interaction was more likely. For the 10 s measurement, the interaction of one, two or three catechol groups with the surface were all of approximately equal probability. Besides the shift to multiple interactions with increased dwell time, it seems that the force of a single catechol–titanium oxide interaction increases with increasing dwell time. The maximum force peaks in Figure 2A are fitted with a Gaussian function and the peak values as well as the standard variation were extracted. These values are plotted in Figure 2D against the dwell time. The forces of the single peak as well as the double peak increased with increasing dwell time. The increase was largest between zero dwell time and 1 s dwell time but there was still some measurable increase in force between 4 s and 10 s dwell time, indicating a slow adhesion process of hPG-catechol on titanium dioxide. This is probably due to the required molecular rearrangement of the hPG in order to properly position the catechol groups for the interaction with the surface.

**Figure 3:**
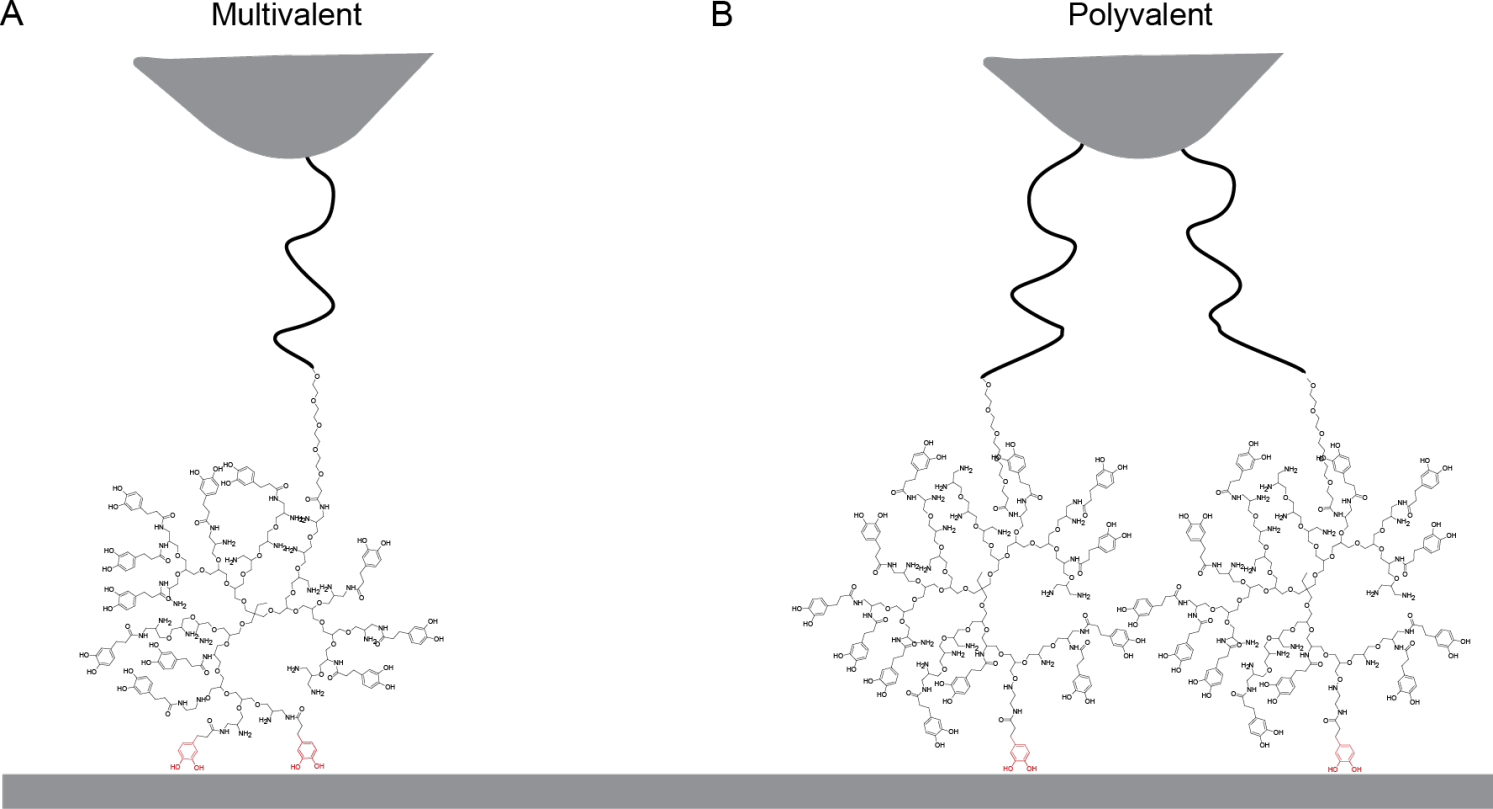
Schematics of the different possibilities for attachment via multiple catechols. A) Multivalent attachment: one hPG molecule is attached to the surface via two catecholic surface anchors. B) Polyvalent attachment: two hPG molecules are attached to the surface with one catechol each and they are attached to the tip via two different PEG linkers.

The double peaks in the measurement could be multivalent, as illustrated in Figure 3A, or polyvalent, as in Figure 3B. In a multivalent interaction, more than one catechol group of the same hPG molecule interacts with the surface. For this to be possible, the orientation of the catechol end groups on the surface must be correct for more than one catechol group. With 40% catechol end groups, this should be possible. The other possibility is that more than one functionalized hPG is covalently attached to the tip and that two hPG molecules can simultaneously interact with the surface.

**Figure 4:**
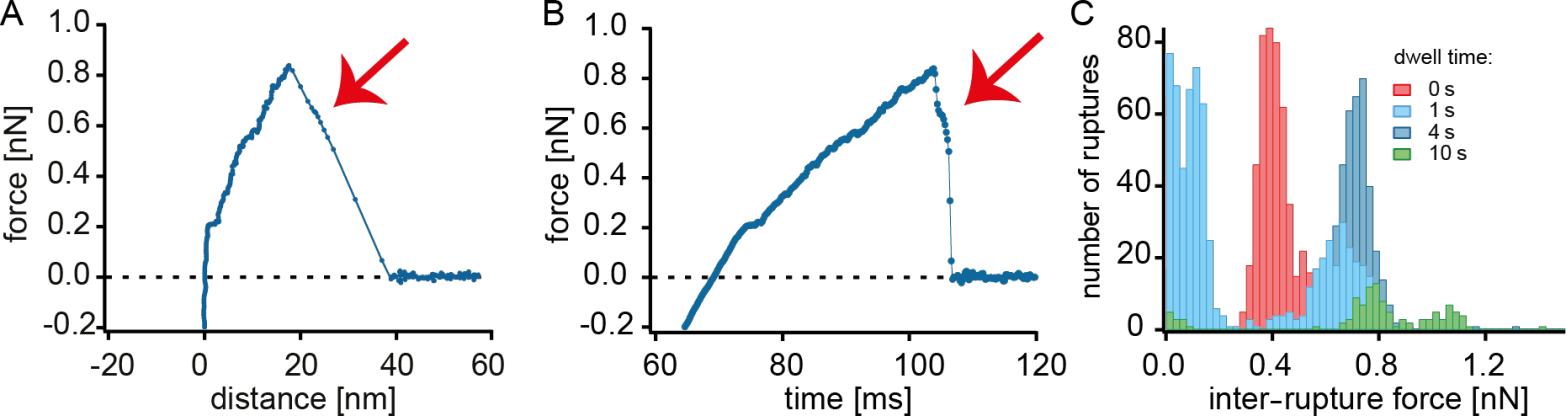
A) Force–distance curves where the rupture is not smooth but rather interrupted by a cluster of measurement points. This inter-rupture force is indicated by a red arrow. B) Same curve as in A) depicted as force vs time. C) Histograms of inter-rupture forces for the different dwell times.

In a number of force–distance curves it was apparent that more than one hPG molecule is part of the interaction. In Figure 4A,B a cluster of measurement points (indicated by a red arrow) related to the rupture of the linker is shown. This cluster of points indicates that there is a second hPG on a different linker and the second hPG catechol bond can hold the force for a short time before rupture. The cluster of measurement points is called an inter-rupture force. In Figure 4C the values of the inter-rupture forces were collected in histograms. Here we ensured that the given inter-rupture force value is the last interaction before the force drops to zero. It is interesting to have a closer look at the inter-rupture forces. They followed the same trend of increasing force with surface contact time but are about 100 pN higher than the maximum forces. The reason for the slightly higher forces is that the force load was shared with another hPG molecule and the full force was only experienced for a short time after the rupture of the first hPG. For the 10 s dwell time measurement, a second peak of inter-rupture force greater than 1 nN was observed. This indicates the presence of one hPG molecule with two catecholic interactions and means that in the case of the triple maximum force peak, two hPG molecules were involved: one with a single catechol anchor and the second with two catecholic anchors. Note that in the last case, two catechols with two PEG linkers were involved that shared the applied force. Therefore, the triple maximum force peak with forces of up to 2 nN could be measured despite rupture forces of 1.4 nN for the Si–O bond between the AFM tip and a single PEG linker.

In summary, a prolonged surface dwell time increased the probability of catecholic interactions. In many of the curves, two different catecholic hPGs interacted simultaneously with the surface in a polyvalent manner as can be seen by the inter-rupture forces. Multivalent binding of two catechols in a single hPG also occurred, but more rarely. In the case of the 10 s dwell measurements there was even a triple interaction involving both poly- and multivalent anchoring. In addition, increasing the surface contact time leads to higher interaction forces for a single catechol on hPG.

## Conclusion

The desorption of different catechol-functionalized hyperbranched polyglycerol molecules from a titanium dioxide surface can lead to very high forces and a reversible bond formation. We described several parameters necessary to obtain reliable, high monovalent desorption forces. In addition, we quantified the poly/multivalency of bonds and showed first steps towards controlling this valency. Notably, a very high percentage (40%) of catechol groups on hPGs must be introduced to obtain di- or trivalent interactions. The data also show that the dwell time of catechols in contact with surfaces is crucial. Dwell times on the timescale of seconds increase not only the probability for higher valency, but also the force per single catechol bond. This underlines that catechols need some time to reach the optimum conformation for interface formation [19], possibly by a “standing up/lying down” mechanism [25] or even more likely via “rolling” into minima of the free energy [26]. We anticipate that these results will help improve catecholic hPGs as stable surface coatings in aqueous buffer [22].

## Experimental

### hPGs

Hyperbranched polyglycerol (hPG) with *M*_n_ ≈5000 g/mol and *M*_w_ ≈7500 g/mol, was polymerized by a one-step, ring-opening, anionic polymerization, as described in the literature [27–28]. Trimethylolpropane (TMP) was used as the initiator. Amine-functionalized hPG was prepared according to previously published procedures [29]. 3,4-Dihydroxyhydrocinnamic acid and acrylic acid molecules were grafted onto the amine groups by amide coupling to introduce catechol groups [22]. Different molecules with different numbers of amine and catechol end groups were prepared, including hPG with 2% amine groups and 8% catechol groups and hPG with 60% amine groups and 40% catechol groups [18].

hPG with 2% amine groups and 8% catechol groups: ^1^H NMR (700 MHz, MeOD) δ 6.70–6.54 (m, 27.44H, CH_arom._), 3.90–3.17 (m, 541.61H, PG-backbone), 2.77 (m, 18.44H, COCH_2_C*H*_2_C), 2.45 (m, 18.51H, COC*H_2_*CH_2_C), 1.41–1.39 (m, 2H, CC*H_2_*CH_3_ of starter), 0.90 (t, 3H, CCH_2_C*H_3_*, of starter) ppm; ^13^C NMR (175 MHz, MeOD) δ 175.95 (C=O), 175.69 (C=O), 146.34–116.45 (C_arom._), 81.70–43.60 (PG backbone), 39.50 (CO*C*H_2_CH_2_C), 37.75 (CO*C*H_2_CH_2_C), 32.64 (COCH_2_*C*H_2_C), 31.95 (COCH_2_*C*H_2_C), 22.14 (C*C*H_2_CH_3_ of starter), 7.09 (CCH_2_*C*H_3_ of starter) ppm.

hPG with 60% amine groups and 40% catechol groups: ^1^H NMR (700 MHz, MeOD) δ 6.72–6.52 (m, 129.12H, CH_arom._), 4.03–2.97 (m, 541.61H, PG-backbone), 2.75 (m, 85.22H, COCH_2_C*H*_2_C), 2.48 (m, 87.09H, COC*H*_2_CH_2_C), 1.49–1.39 (m, 2H, CC*H_2_*CH_3_ of starter), 0.90 (t, 3H, CCH_2_C*H_3_*, of starter) ppm; ^13^C NMR (175 MHz, MeOD) δ 176.86 (C=O), 176.29 (C=O), 146.31–111.87 (C_arom._), 81.18–52.87 (PG backbone), 39.13 (CO*C*H_2_CH_2_C), 37.57 (CO*C*H_2_CH_2_C), 32.31 (COCH_2_*C*H_2_C), 31.30 (COCH_2_*C*H_2_C), 24.46 (C*C*H_2_CH_3_ of starter), 7.26 (CCH_2_*C*H_3_ of starter) ppm.

### TiO_2_ surface

TiO_2_ slides were prepared by sputtering titanium onto silicon wafers. The sputter process was performed using a commercially available radio frequency magnetron sputter unit (Edwards Auto 306). The purity of the Ti target was 99.995%. The titanium was deposited with a power of 83 W for 4 min. The surface layer was naturally oxidized. Directly before the AFM measurements, the TiO_2_ slides were put in an oxygen plasma (100 W, 0.3 mbar, 1 h, Edwards GMBH, Kirchheim, Germany) and afterwards rinsed with ultrapure water.

### Tip functionalization

The molecule is functionalized to the tip through covalent bonds in a similar manner as previously described [2]. Silicon nitride cantilevers (MLCT, Bruker SPM probes, Camarillo, USA) were first activated in an oxygen plasma chamber (20 W, 0.3 mbar) for 15 min. The cantilevers were rinsed with dry acetone (VWR, Germany) and then incubated for 10 min in a Vectabond (Axxora, Germany) solution (50 µL Vectabond in 2.5 mL dry acetone) for silanization. Afterwards they were rinsed in dry acetone and dry chloroform (VWR, Germany). PEG–Di–NHS (10 kDa, Rapp Polymere GmBH, Tübingen, Germany) was dissolved in dry chloroform (2.5 mM) and the cantilevers were incubated for 60 min. The cantilevers were then rinsed in dry chloroform, ethanol and in the probe molecule reaction buffer and incubated for 1 h in 1 mg/mL probe molecule solution. Dopamine (Sigma-Aldrich) and the hPG without catechol groups were dissolved in sodium borate buffer (50 mM, pH 8.1). hPGs with catechol end groups were dissolved in dry methanol (VWR, Germany). The strength of the Si–O bond is the weakest link of the functionalization and fails at approximately 1.4 nN [16].

### AFM measurements

The AFM force spectroscopy measurements were carried out with an MFP-3D device (Oxford Instruments) equipped with a fluid cell at room temperature. The measurements were performed in double distilled water, PBS and McIlvaines buffer at pH 3. For each measurement the Inverse Optical Lever Sensitivity (InvOLS) of the functionalized cantilever was determined from the indentation slope and the spring constant calibrated with the thermal noise method according to [30]. The tip velocity was 1 µm/s and the standard dwell time was 1 s.

### Data analysis

The data handling and analysis was performed in Igor Pro (Wave Metrics). Force curves were automatically analyzed for interaction events and the maximum detachment force (maximum force) extracted. To exclude nonspecific effects from the tip–surface interaction, events closer than 15 nm to the surface were excluded.
